# Detection of oxalyl-CoA decarboxylase (*oxc)* and formyl-CoA transferase (*frc)* genes in novel probiotic isolates capable of oxalate degradation in vitro

**DOI:** 10.1007/s12223-024-01128-5

**Published:** 2024-01-13

**Authors:** HebatAllah Ibrahim AbdElazeim Youssef

**Affiliations:** Microbiology Department, Faculty of Science, Ain Shams University, El-Khalyfa El-Mamoun st. Abbasya, Cairo, 11566 Egypt

**Keywords:** Oxalate, Degradation, Probiotic, LAB, Oxalyl-CoA decarboxylase, Formyl-CoA transferase

## Abstract

Oxalate degradation is one of lactic acid bacteria’s desirable activities. It is achieved by two enzymes, formyl coenzyme A transferase (*frc*) and oxalyl coenzyme A decarboxylase (*oxc*). The current study aimed to screen 15 locally isolated lactic acid bacteria to select those with the highest oxalate degradation ability. It also aimed to amplify the genes involved in degradation. MRS broth supplemented with 20 mM sodium oxalate was used to culture the tested isolates for 72 h. This was followed by an enzymatic assay to detect remaining oxalate. All isolates showed oxalate degradation activity to variable degrees. Five isolates demonstrated high oxalate degradation, 78 to 88%. To investigate the oxalate-degradation potential of the selected isolates, they have been further tested for the presence of genes that encode for enzymes involved in oxalate catabolism, formyl coenzyme A transferase (*frc*) and oxalyl coenzyme A decarboxylase (*oxc*). Three strains showed bands with the specific OXC and FRC forward and reverse primers designated as (SA-5, 9 and 37). Species-level identification revealed *Loigolactobacillus bifermentans*, *Lacticaseibacillus paracasei*, and *Lactiplantibacillus plantarum*. Preliminary results revealed that the tested probiotic strains harbored both *oxc* and *frc* whose products are putatively involved in oxalate catabolism. The probiotic potential of the selected strains was evaluated, and they showed high survival rates to both simulated gastric and intestinal fluids and variable degrees of antagonism against the tested Gram-positive and negative pathogens and were sensitive to clarithromycin but resistant to both metronidazole and ceftazidime. Finally, these strains could be exploited as an innovative approach to establish oxalate homeostasis in humans and prevent kidney stone formation.

## Introduction

Oxalate is a very common component of the human diet (fruits, vegetables, legumes, grains, coffee, chocolate, and nuts), which can accumulate in renal tissue and bind calcium to form calcium oxalate crystals. In food, oxalic acid is typically found as sodium or potassium oxalate, which is water soluble, or calcium oxalate, which is insoluble (Liebman and Al-Wahsh 2011). Both active and passive uptake mechanisms are utilized for oxalate absorption throughout the gastrointestinal tract. After oxalate ingestion, the peak recovery is between 2 and 6 h. The absorption could be either in the stomach (where gastric acidic pH increases the solubility of food-derived oxalate, making the ion available for absorption) or in the small intestine (Liebman and Al-Wahsh 2011). Inflammation caused by calcium oxalate crystal accumulation, along with innate or secondary renal changes, could significantly affect renal function (Turroni et al. 2010). About 80% of kidney stones are predominantly composed of calcium oxalate (Worcester and Coe 2010). Available treatments for patients with calcium oxalate stone diseases are limited and rarely sufficient for the reduction of urinary oxalate excretion (Sasikumar et al. 2013). Unfortunately, an oxalate-free diet is difficult to achieve and deficient in essential micronutrients. After discovering oxalate-degrading bacteria in the human gastrointestinal tract, an alternative approach to reducing urinary oxalate has emerged (Kaufman et al. 2008). Although the direct cause of kidney stone formation is not known, reports suggest it is probably a multifactorial disease (Hornberger and Bollner 2018). The presence of oxalate-degrading bacteria in the human gut microbiome is essential as humans lack the enzymes required to metabolize endogenous and dietary oxalate (Mehra and Viswanathan 2021). Studies show that degradation of oxalate by intestinal bacteria decreases oxalate absorption leads to a reduction in urinary oxalate levels and decreases stone formation (Zhao et al. 2017). Novel oxalotrophic bacteria are being increasingly studied as potential probiotic candidates directed toward kidney stone diseases (Ghate et al. 2021). Microorganisms that contribute to oxalate degradation within the gut include *Oxalobacter formigenes* and *Lactobacillus acidophilus* (Weese et al. 2004). *O. formigenes* is a non-pathogenic Gram-negative anaerobic bacterium, which has been demonstrated to be present in the colon of some, but not all adults, and has well-established oxalate-degrading capabilities (Duncan et al. 2002). Oxalic acid catabolism by *O. formigenes* involves three enzymes: formyl-CoA transferase (*frc*), which activates an oxalate molecule to oxalyl-CoA (Jonsson et al. 2004); oxalyl-CoA decarboxylase (*oxc*), which decarboxylates the oxalyl-CoA molecule to formyl-CoA (Berthold et al. 2005); oxalate: formate antiporter, which catalyzes the exchange of extracellular oxalate and intracellular formate (Wang et al. 2006). *O. formigenes* is not an ideal probiotic due to its antibiotic sensitivity and low pH (Wigner et al. 2022). Most anaerobic bacteria metabolize oxalate to carbon dioxide and formate. Formate is further oxidized by dehydrogenases. The probiotic administration of *Lactobacillus* and *Bifidobacterium* species has also been associated with a significant reduction in urine oxalate levels in both animals and humans (Okombo and Liebma 2010). More than 200 species and subspecies of *Lactobacillus* have been formally recognized (Salvetti et al. 2012). This work tested the oxalate-degrading ability of various LAB isolates and confirmed their activity by detecting (*oxc)* and (*frc)* genes involved in oxalate degradation. Also, the probiotic potential of the chosen isolates was assessed.

## Materials and methods

### Isolation of lactic acid bacteria

Using sterile cups, ten raw milk samples were collected from dairy farms near Cairo and immediately carried in an ice box to the lab. In there, the samples were bacteriologically cultivated by making serial dilutions by adding 1 mL of the sample to 10 mL of sterile peptone water. Following homogenization, each sample was diluted 10^−5^ and placed on MRS agar medium. The plates were incubated under anaerobic conditions for 48 h at 37 °C. Thirty-seven colonies (SA-1 to SA-37) of various LAB morphologies were selected and streaked on MRS agar medium for further purification. Fifteen Gram-positive colonies with catalase-negative reaction were transferred to MRS broth and kept in the refrigerator at 4 °C for further investigations (Pundir et al. 2013).

### In vitro testing of LAB isolates for oxalate degradation

Fifteen probiotic isolates were tested for oxalate degradation activity according to Cho et al. (2015). A sterile MRS broth was supplemented with a filter-sterilized 20-mM sodium oxalate solution. The MRS-oxalate solution consisted of 4.75 mL of sterilized MRS broth and 4.75 mL of filter-sterilized sodium oxalate solution. Fresh cultures of the tested bacterial isolates were prepared by inoculation into pure MRS broth and incubated anaerobically at 37 °C for 24 h. Then, (500 µL) of each isolate was inoculated into the prepared MRS-oxalate broth and incubated anaerobically at 37 °C for 72 h. Negative control samples were prepared without inoculation. After the incubation period, all samples were centrifuged at 7000 × g for 20 min to pellet the bacteria. After filter sterilization (0.22 µm filter), pH was measured, and supernatants were stored in the freezer until oxalate was quantified.

### Colony counts

Ten-fold serial dilutions were made from each isolate from both MRS and MRS-oxalate broths. A 100 µL aliquot of each diluent was inoculated and spread onto MRS plates and incubated anaerobically at 37 °C for 48 h. Growth was assessed by counting colonies on plates.

### Detection of residual oxalate

Oxalate concentrations in the culture supernatants were measured in triplicate using an oxalate enzymatic experiment kit (Catalog # K663-100; Biovision, Milpitas, USA), based on oxalate oxidation by oxalate oxidase. Oxalate assay protocol according to manufacturer’s instructions:

1. Fifty microliters of each broth sample was added to a 96-well plate.
2. Standard curve preparation: oxalate standard was diluted to 1 mM (1 nmol/µL) by adding 10 µL of 100 mM oxalate standard to 990 µL dH_2_O, mixed well. Then, 0, 2, 4, 6, 8, and 10 µL of the 1 mM oxalate standard were added to a series of wells in a 96-well plate. Then, volume was adjusted to 50 µL/well with oxalate assay buffer to generate 0, 2, 4, 6, 8, and 10 nmol/well of oxalate standard.
3. Two microliters of oxalate converter was added to each standard and sample well and then mixed and incubated at 37 °C for 1 h.
4. For each well, 50 µL of the reaction mix containing (oxalate development buffer 46 µL oxalate enzyme mix 2 µL, and oxalate probe 2 µL) was added to each well and then mixed well.
5. The reaction was then incubated at 37 °C for 60 min. Finally, the absorbance was measured at 450 nm.
6. Calculations: The oxalate standard reading was subtracted from all readings to plot the oxalate standard curve.
  The corrected sample reading was applied to the standard curve to get B nmol of oxalate amount in the sample wells. Sample oxalate concentration (*C*) = *B*/*V*×*D* = nmol/mL = µM, where *B* is the amount of oxalate in the sample well from the standard curve (nmol). *V* is the sample volume used in the reaction well (mL). *D* is the sample dilution factor.

### Detection of oxalyl-CoA decarboxylase (*oxc*) and formyl-CoA Transferase (*frc*) genes in isolates

For DNA extraction, 200 µL of each sample (liquid media that contains bacteria) was placed in a microcentrifuge tube and added 95 µL water, 95 µL solid tissue buffer (blue), and 10 µL proteinase K and then mixed thoroughly and incubated at 55 °C for 2 h. Mix thoroughly and centrifuge at 12,000 × g for 1 min. Aqueous supernatants were transferred to a clean tube (300 µL). Afterwards, 600 µL of genomic binding buffer was added and thoroughly mixed. The mixture was then transferred to a Zymo-Spin^™^ IIC-XL column in a collection tube. Centrifuged at 12,000 × g for 1 min, the collection tube was discarded with the flow through. Four-hundred-microliter DNA Pre-Wash Buffer was added to the column in a new Collection Tube and centrifuged at (12,000 × g) for 1 min. Then, 700-µL g-DNA Wash Buffer was added and centrifuged at (12,000 × g) for 1 min. Empty the collection tube. Two hundred microliters of g-DNA wash buffer was added and centrifuged at 12,000 × g for 1 min. The collection tube was discarded. Thirty-microliter elution buffer was added and incubated for 5 min, then centrifuged at (12,000 × g) for 1 min. Then, PCR was carried out using primers designed for the *oxc* and *frc* genes from *Lactobacillus acidophilus* NCFM according to Altermann et al. (2005).

The primer sets are as follows:

- *oxc*-L (5′-CTTGAAATGCAAGATGAAAGCA-3′)
- *oxc*-R (5′-CTTCAGTCATTATTTATTCTCC3′)
- *frc*-L (5′-GGAGAATAAATAATGACTGAAGA-3′)
- *frc*-R (5′-CGGTAAAAATTAATTATTCACC-3′)

The PCR amplification program for each primer is described in Tables 1 and 2.

**Table 1:** Temperature and times for the PCR protocol for *oxc* primer

**Stage**	**Temperature**	**Time**
1	95 °C	5 min
2.1	95 °C	30 s
2.2	Primer Tm 44 °C	30 s
2.3	72 °C	30 s
Repeat stage 2 for 40 cycles		
3	72 °C	5 min

**Table 2:** Temperature and times for the PCR protocol for *frc* primer

**Stage**	**Temperature**	**Time**
1	95 °C	5 min
2.1	95 °C	30 s
2.2	Primer Tm 46 °C	30 s
2.3	72 °C	30 s
Repeat stage 2 for 40 cycles		
3	72 °C	5 min

Finally, after gel electrophoresis and band purification, sequencing of the PCR purified products was performed at GATC Biotech (Germany Company) using ABI 3730xl DNA sequencer by using forward and reverse primers.

### Probiotic properties of the selected isolates

#### Tolerance of isolates to simulated gastric and intestinal fluids

To examine LAB isolates’ tolerance, they were exposed to simulated gastric fluid (SGF) and simulated intestinal fluid (SIF). One-microliter aliquots (approximately 7 log_10_ CFU/mL) of each tested LAB isolates suspension were inoculated in 10 mL of PBS with pH adjusted to 2.5 (using 1 M HCl) incorporated with 3 g/L pepsin or supplemented with 3% (w/v) ox-gall and 1 g/L pancreatin adjusted by 0.1 M NaOH to (pH 7.4). Then, cells were incubated aerobically at 37 °C for 3 h. After incubation, 1 mL aliquots from each system were taken, serially diluted in sterile peptone water (10^−1^–10^−5^), and then plated onto MRS agar to count live cells. After 48 h of anaerobic incubation at 37 °C, viable cells were counted, and the results were represented as (log CFU/mL). For the controls, LAB were grown in PBS at pH 7.2 (Monteagudo-Mera et al. 2012).

#### Antagonistic activity against pathogens

According to Jacobsen et al. (1999), spot agar was used to assess the antagonistic activity of lactic acid bacteria against various foodborne pathogenic bacteria and clinical isolates. LAB were grown overnight in MRS broth and spotted onto MRS agar containing 0.2% (w/v) glucose and 12 gm/L agar and incubated anaerobically for 24 h at 37 °C. A 100 µL aliquot of each indicator bacterium suspension was then mixed with 10 mL of soft nutrient agar (7 g/L agar) and poured over the spot-inoculated MRS agar. The plates were incubated aerobically at 37 °C for 48 h. The antagonistic activity was recorded as the diameter (mm) of the growth inhibition zones around each spot. Positive inhibitory activity was defined as a growth inhibition zone with a diameter greater than 1 mm surrounding the spot.

#### Antibiotic susceptibility

Antibiotic susceptibility of the selected strains was assayed using the drug disc agar diffusion method (Bauer et al. 1966). Two hundred microliters (10^7^ CFU/mL) of cultured 24-h strain suspension was spread over the MRS plates. In this study, ten commercial antibiotics (OXOID) were selected, including meropenem (10 µg), metronidazole (5 µg), clarithromycin (15 µg), piperacillin/tazobactam (110 µg), ceftazidime (30 µg), levofloxacin (5 µg), amoxicillin (10 µg), ciprofloxacin (5 µg), ampicillin (10 µg), and cefepime (30 µg), and were placed on the above MRS agar medium. The diameter of the clear zone around the discs was measured after incubation at 37 °C under anaerobic conditions for 48 h. Resistance and sensitivity were expressed according to the American Clinical and Laboratory Standards Institute guidelines (CLSI 2020).

## Results

**Fig. 1:**
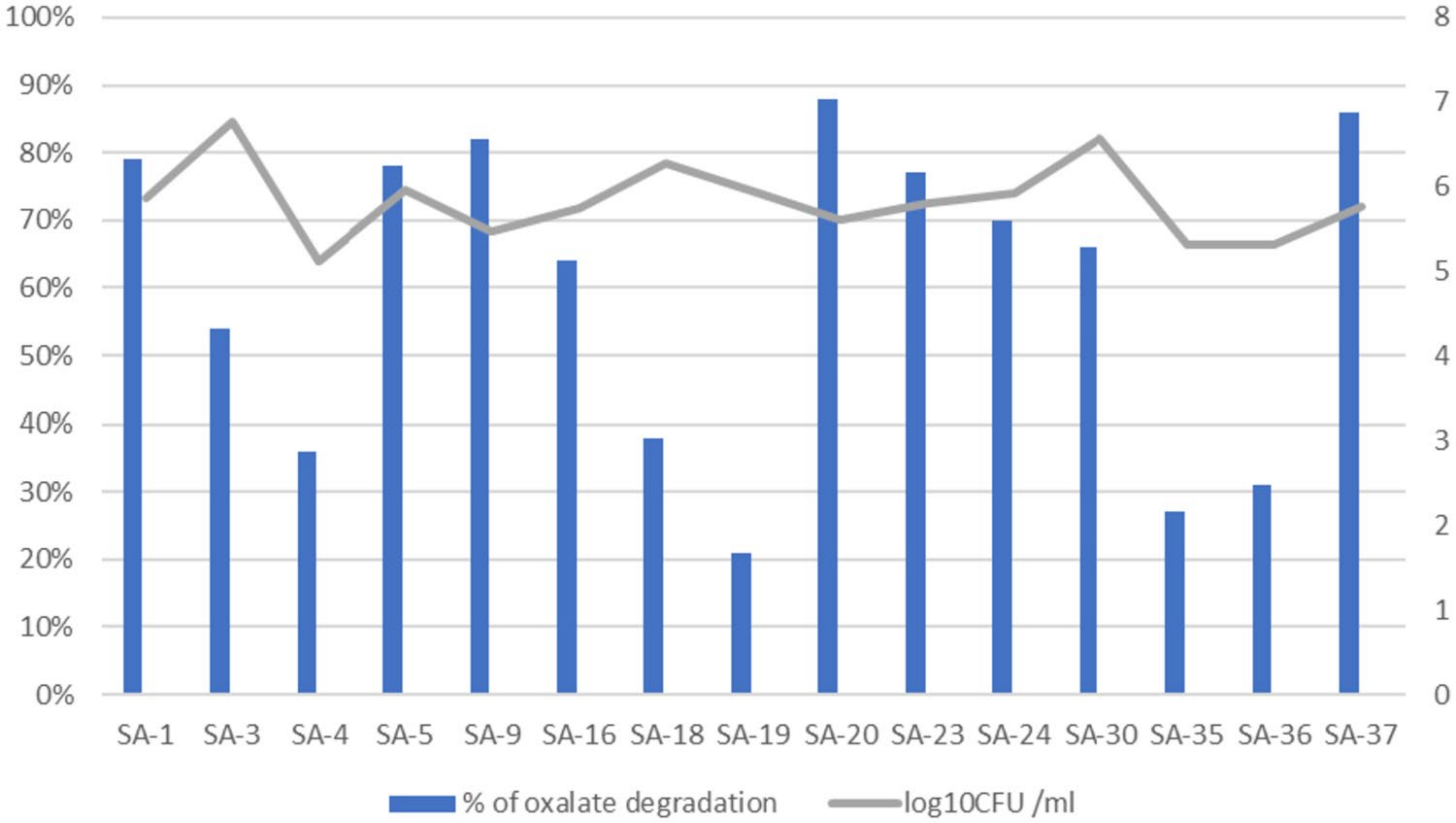
Oxalate degradation activity by the tested LAB isolates

In this study, fifteen isolates were examined for their oxalate degradation ability. Screening was done by enzymatic assay. All isolates could grow and degrade 20 mM sodium oxalate, but to variable degrees. Significant oxalate degradation was observed in ten isolates, which utilized more than 50% of oxalate in broth medium. As shown in Table 3 and Fig. 1, oxalate degradation % ranged from 21 to 88%. In particular, isolate number SA 20 showed the highest oxalate-degrading activity while isolate SA 19 showed the lowest activity. Also, the pH of the supernatant after incubation decreased from 6.03 for control to 4.02 and up to 5.68. Colony counts of isolates after 72 h of incubation ranged from 5.11 to 6.77 log_10_/ CFU.

**Table 3:** Oxalate degrading activity of LAB isolates, pH of culture broth, and colony counts

**SA-code**	**absorbance**	**Concentration (nM)**	**Concentration (mg/100 mL)**	**% of oxalate degradation**	**pH**	**log**_**10**_**CFU /mL**
**Control**	0.612	3605.63	48.333	_	6.03	_
**SA-1**	0.208	760.56	10.195	79%	5.14	5.86
**SA-3**	0.336	1661.97	22.278	54%	4.02	6.77
**SA-4**	0.426	2295.77	30.774	36%	5.52	5.11
**SA-5**	0.213	795.77	10.667	78%	5.46	5.96
**SA-9**	0.188	619.72	8.307	82%	5.22	5.47
**SA-16**	0.282	1281.69	17.181	64%	4.22	5.74
**SA-18**	0.416	2225.35	29.830	38%	4.32	6.27
**SA-19**	0.501	2823.94	37.854	21%	4.38	5.93
**SA-20**	0.161	429.58	5.758	88%	5.63	5.60
**SA-23**	0.217	823.94	11.045	77%	5.45	5.80
**SA-24**	0.252	1070.42	14.349	70%	4.48	5.92
**SA-30**	0.272	1211.27	16.237	66%	5.49	6.57
**SA-35**	0.475	2640.85	35.400	27%	5.48	5.30
**SA-36**	0.451	2471.83	33.134	31%	5.68	5.30
**SA-37**	0.172	507.04	6.797	86%	4.40	5.77

### Detection of (*oxc*) and (*frc*) genes

**Fig. 2:**
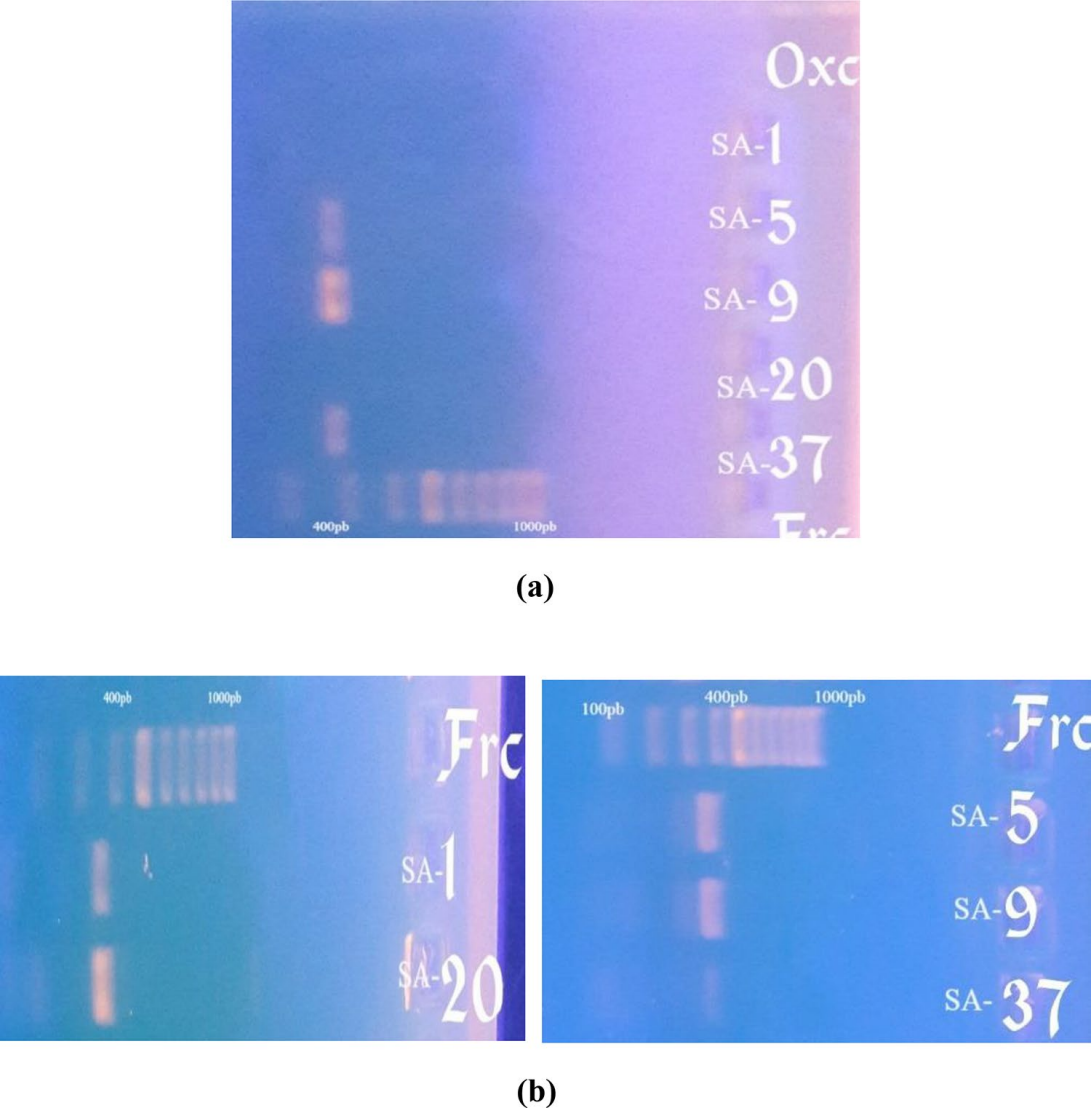
**a** Bands of the amplified (*oxc*) gene and **b** bands of the amplified (*frc*) gene from the selected oxalate degrading probiotic bacterial strains

According to the oxalate degradation activity results, five isolates with the highest activity were selected for further genetic analysis. These isolates were examined for the (*oxc)* and (*frc)* genes encoding the oxalyl-CoA decarboxylase and the formyl-CoA transferase involved in oxalate catabolism. Among the five isolates (SA 20, SA 37, SA 9, SA 1, and SA 5) chosen to be cloned and sequenced, only three isolates (SA 37, SA 9, and SA 5) exhibited the presence of (*oxc*) and (*frc*) genes, while the other two (SA 20 and SA 1) isolates showed bands only for the (*frc)* gene as shown in Fig. 2a, b.

Amplified bands of both genes from the three isolates were excised and gel purified. These isolates were chosen based upon the intensities of the bands on the gel in Figure. The sequences were aligned with published sequences of the type strains from GenBank. Sequence identity revealed 100% homologies with the strains shown in Table 4. The obtained sequences were deposited in the GenBank database under accession numbers PRJNA790634, PRJNA790635, and PRJNA790636.

**Table 4:** Isolates identification and accession numbers

**Isolate no.**	**Identification**	**Similarity %**	**Accession number**
**SA 5**	***Loigolactobacillus bifermentans***	100%	PRJNA790634
**SA 9**	***Lacticaseibacillus paracasei***	100%	PRJNA790635
**SA 37**	***Lactiplantibacillus plantarum***	100%	PRJNA790636

### Sequence alignments and phylogenetic inference

**Fig. 3:**
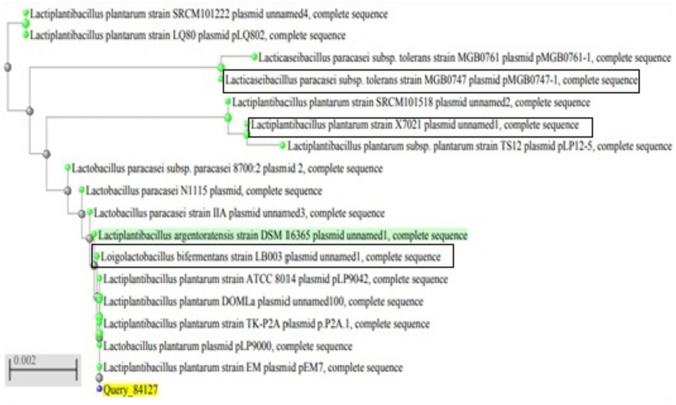
Phylogenetic tree showing the relative positions of isolates and similar strains as inferred by the neighbor-joining method of (*oxc)* and (*frc)* gene sequences

Phylogenetic analysis of the identified strains and similar strains was performed based on *oxc* and *frc* genes. A phylogenetic tree was constructed by the neighbor-joining method using NCBI blast website. The strains examined were divided into three groups as shown in Fig. 3. All are included in the family Lactobacillaceae.

#### Probiotic properties

##### Tolerance of selected isolates to simulated gastric and intestinal fluids

To determine tolerance to acid and bile salt, each of the three selected isolates was subjected to SGF and SIF for 3 h (Table 5). All isolates showed an excellent survival rate (over 90%) in simulated gastric fluid with a slight reduction of viable counts ranging from 0.6 to 0.7 log_10_ cycle compared to control (pH 7.2). On the other hand, after 3 h incubation in simulated intestinal fluid with 3% (w/v) ox-gall, isolates exhibited good tolerance with survival rates ranging from 77 to 86%.

**Table 5:** Viable counts and survival rates of LAB isolates (log_10_ CFU/mL) after exposure to simulated gastric and intestinal fluids for 3 h

**Isolate**	**Control, Log**_**10**_ **CFU/mL**	**SGF, Log**_**10**_ **CFU/mL**	**Survival rate %**	**SIF, Log**_**10**_ **CFU/mL**	**Survival rate %**
**SA-5**	7.9	7.2	91%	6.1	77%
**SA-9**	7.4	6.7	90%	6.4	86%
**SA-37**	7.7	7.1	92%	6.5	84%

#### Antagonistic activity against pathogens

The three selected isolates based on their oxalate degrading activity were tested for their antagonistic potential toward various Gram-positive and Gram-negative indicator pathogenic bacteria (Table 6). All isolates showed variable degree of inhibition toward all tested pathogens although isolate SA-9 did not show an inhibition zone against *S. aureus.* The three isolates strongly inhibited *B. cereus*, moderately inhibited *S. typhimurium*, and weakly suppressed *K. pneumonia.*

**Table 6:** Antagonistic activity of the three selected LAB isolates against pathogenic bacteria

**Pathogen**	**LAB isolate**		
	**SA-5**	**SA-9**	**SA-37**
***Bacillus cereus* B-3711**	**+++ **	**+++ **	**+++ **
***Listeria monocytogenes* type I**	**++ **	**+++ **	**+++ **
***Pseudomonas sp.***	**++ **	**++ **	**+++ **
***Staphylococcus aureus***	**++ **	**-**	**+ **
***Helicobacter pylori***	**++ **	**+ **	**+ **
***Escherichia coli***	**++ **	**++ **	**+++ **
***Yersinia enterocolitica* ATCC 9610**	**++ **	**++ **	**+ **
***Salmonella typhimurium* ATCC 14028**	**++ **	**++ **	**++ **
***Klebsiella pneumoniae***	**+ **	**+ **	**+ **

− no inhibition, + 4–8 mm, ++ 8–12 mm, +++ ≥ 20 mm

#### Antibiotic susceptibility

Table 7 shows the antibiotic sensitivity profile of the three selected LAB. The three isolates were sensitive to clarithromycin. They were resistant to metronidazole and ceftazidime. Moreover, isolate SA-9 was sensitive to levofloxacin and amoxycillin, while isolate SA-5 showed sensitivity to the piperacillin/tazobactam combination. Finally, isolate SA-37 exhibited cefepime sensitivity.

**Table 7:** Antibiotics susceptibility test of the selected LAB isolates toward some antibiotics

	**Antibiotics**									
**Isolate**	**Meropenem (MEM) 10µL**	**Metronidazole (MTZ) 5µL**	**Piperacillin/Tazobactam (TZP) 110µL**	**Ampicillin (AM) 10µL**	**Amoxicillin (AML) 10µL**	**Cefepime (FEB) 30µL**	**Levofloxacin (LE) 5µL**	**Ciprofloxacin (CIP) 5µL**	**Clarithromycin (CLR) 15µL**	**Ceftazidime (CAZ) 30µL**
**SA-5**	R	R	S	I	R	R	R	R	S	R
**SA-9**	I	R	R	R	S	R	S	I	S	R
**SA-37**	R	R	R	R	R	S	R	R	S	R

## Discussion

Daily uptake of oxalate (> 45 mg) from food and/or synthesized in the liver may stimulate kidney stone formation and other health problems linked to high levels of urinary oxalate (Von Unruh et al. 2004). During aerobic growth, the gastrointestinal tract microbiota, such as biodegrading probiotic bacteria, can metabolize oxalate to CO_2_ and formate. Colonization of the intestine with highly efficient, oxalate-degrading intestinal probiotic bacteria may reduce the risk of calcium oxalate stone disease. The objectives of the present study were to determine the oxalate degradation activity of 15 lactic acid probiotic isolates. This was done by quantitative analysis of residual oxalate amount in the supernatants of bacterial cultures previously cultured on MRS with 20 mM sodium oxalate. Results revealed that evaluated isolates showed high oxalate degrading ability as the amount of the residual sodium oxalate in culture broth ranged from 5.7 to 37 mg/100 mL. In accordance with Giardina et al. (2014), *L. plantarum* and *L. acidophilus* displayed the maximum oxalate-degrading activity (70% and 59.2%, respectively). Similar results in a previous study by Hatch (2017) that evaluated the potential of two probiotics (*L. acidophilus and L. gasseri*) to exert oxalate degradation through liquid scintillation demonstrated significant degradation by both species. *L. acidophilus* showed 100% degradation of ^14^C-oxalate with ~44% of counts remaining representing ^14^C-formate in the media from enzymatic ^14^C-oxalate degradation via oxalate decarboxylase (Cho et al. 2015). Also, Darilmaz et al. (2019) reported that oxalate degradation by *L. fermentum* IP5 was 38.18 and 29.60% after growth in 10 mM and 20 mM MRS-ox plus 5% inulin media, respectively.

Intestinal microbiota, such as lactic acid bacteria, degrade oxalic acid. Some *Lactobacillus* spp., such as *L. rhamnosus*, *L. casei*, and *L. gasseri*, have oxalate-degrading enzymes, such as formyl-CoA transferase (*frc*) and oxalyl-CoA decarboxylase (*oxc*) (Lewanika et al. 2007). The current study attempted to amplify the genes coding for formyl coenzyme A transferase (*frc*) and oxalyl coenzyme A decarboxylase (*oxc*) enzymes to assess oxalate degradation by the tested isolates. Three of the tested isolates showed band size (~400 bp) with primers of both enzymes which were also confirmed by the oxalate degradation experiment. Similar previous reports were obtained by Ellis et al. (2015); they isolated DNA from two probiotic supplements (*Lactobacillus plantarum* and *Lactococcus lactis* subsp. *lactis*) and performed PCR using primers designed to detect *oxc* in *Lactobacillus* strains that amplify the oxalate decarboxylase gene and found that both probiotics exhibited a band of the expected size (419 bp). Additionally, Mehra and Viswanathan (2021) found that *L. paragasseri* UBLG-36 encoded enzymes involved in oxalate catabolism, which degraded oxalate by more than 45% in vitro. Similar results were obtained by Miller et al. (2014) who amplified *oxc* gene from nine isolates (four isolates of *Lactobacillus gasseri*, one isolate of *Lactobacillus animalis*, two isolates of *Lactobacillus johnsonii*, and two isolates of *Lactobacillus reuteri*), all *Lactobacillus* isolates exhibited a band of the expected size (400 bp). On the other hand, Kullin et al. (2014) investigated and compared the functionality of the FRC gene in parent and mutant (*frc* ^−^) *Lact. reuteri* 100-23C and concluded that the presence of *oxc* and FRC genes do not ensure oxalate degradation in RLF mice under the conditions tested. But the FRC gene product was significant during host digestive tract colonization and survival of acid stress by the tested strain. This is because the loss of the (*frc)* gene led to reduced gut survival levels. Chamberlain et al. (2019) investigated the metabolic profiles of *Lactobacillus acidophilus* and *L. gasseri* by measuring in vitro ^14^C-oxalate consumption and confirming their ability to degrade oxalate even in the presence of other carbon sources, providing support for the use of these *Lactobacillus* species as probiotic treatments for oxalate stone disorder. Finally, the lack of uniformity in oxalate degradation is attributed to both species-to-species and strain-to-strain variations, as evidenced by multiple studies (Azcarate-Peril et al. 2008). Based on sequence similarity, the three selected isolates have been identified as *Loigolactobacillus bifermentans*, *Lacticaseibacillus paracasei*, and *Lactiplantibacillus plantarum* with 100% sequence similarity.

The initial screening and selection of probiotics include testing of the phenotype and genotype stability; survival under harsh conditions in the gastrointestinal tract; protein and carbohydrate utilization patterns; production of antimicrobial substances; antibiotic resistance patterns; and ability to inhibit known pathogens, spoilage organisms, or both (Harzallah and Belhadj 2013). Based upon oxalate degradation activity and detection of (*oxc)* and (*frc)* genes, three LAB were selected and subjected to further assessment of their probiotic properties. This included tolerance to simulated gastric and intestinal fluids, antagonism against some indicator food-borne pathogens and clinical pathogens, and antibiotic sensitivity test. The findings of the current study indicated that the three selected LAB isolates can withstand harsh GI conditions. They have survival rates over 90% for SGF and up to 86% for SIF after 3 h. Similarly, Abonee et al. (2023) studied the tolerance of ten LAB isolates using freshly prepared gastric and intestinal juices and found a non-significant reduction in viable counts (by 1–2 log_10_) after 180 min of treatment, showing good tolerance. Another supporting study by Chouraddi et al*.* (2023) After three hours of exposure, the count (Log_10_ CFU/mL) of the isolates at acidic pH 2 and 0.3% ox-gall. Out of 37 isolates, 17 showed a log reduction in acidic conditions (pH 2) and 11 in bile salt conditions. Moreover, the viability of *L. reuteri* B2 was assessed at pH 2.5 and viability decreased slowly, 6.22 log_10_ CFU/mL (78%) (Popović et al. 2021). Studying the antagonistic activity of the three selected oxalate-degrading LAB proved their potential against all tested food-borne and clinical isolate pathogenic bacteria with varying degrees; the only exception was isolate SA-9 which did not show any inhibition zone toward *Staph. aureus.* Those results were also supported by the study of Garcia et al. (2016) using the agar spot test; all tested *Lactobacillus* strains exhibited antagonism against *Staphylococcus aureus*, *Salmonella typhimurium*, *Salmonella enteritidis*, *Listeria monocytogenes*, and *Escherichia coli* and presented variable susceptibility to different antibiotics. Similarly, Prabhurajeshwar and Chandrakanth (2017) study the antagonistic activity of 16 *Lactobacillus* isolates and the inhibition zones against *Staph. aureus* ranged from 14 to 30 mm, *P. aeruginosa* were 12–25 mm, *E. coli* were 14–28 mm, and for *K. pneumoniae* were 14–26 mm. Resistance toward ampicillin, oxacillin is denoted by the three *Lactobacillus* isolates (T2, T4, and T16) (Prabhurajeshwar and Chandrakanth 2017).

## Conclusions

Previous research found that manipulating the gut flora with the correct probiotic bacteria could improve gastrointestinal tract oxalate levels and decrease oxalate absorption. Finally, in this study, three efficient oxalate-degrading LAB were found. Their safety analyses indicate that they could be promising probiotic candidates for preventing hyperoxaluria. The function and significance of their oxalyl-CoA decarboxylase and formyl-CoA transferase in oxalate catabolism were demonstrated in vitro. This interesting property suggests the potential use of those strains for oxalate degradation in the human gut. Finally, further in vivo studies may help in the development of biological treatments for hyperoxaluria and preventive oxalate stone formation.
